# Factors associated with failure to screen for syphilis during antenatal care in Ghana: a case control study

**DOI:** 10.1186/s12879-015-0868-1

**Published:** 2015-03-13

**Authors:** Edward Tieru Dassah, Yaw Adu-Sarkodie, Philippe Mayaud

**Affiliations:** Department of Obstetrics and Gynaecology, Komfo Anokye Teaching Hospital, P. O. Box KS 1934, Kumasi, Ghana; School of Public Health, Kwame Nkrumah University of Science and Technology, Kumasi, Ghana; School of Medical Sciences, Kwame Nkrumah University of Science and Technology, Kumasi, Ghana; London School of Hygiene and Tropical Medicine, London, UK

**Keywords:** Syphilis screening, Antenatal care, Point of care tests, Ghana

## Abstract

**Background:**

There is little data regarding the effect of ramping up new screening interventions on their uptake by target populations into routine care services in developing countries. This study aimed to determine patient-level factors associated with failure of pregnant women to get screened for syphilis during antenatal care, in the context of a national rollout of rapid syphilis point of care tests (POCTs) in Ghana.

**Methods:**

An unmatched 1:2 case control study conducted among women admitted for delivery in two district hospitals in the Ashanti Region of Ghana from August to October 2010, 7 to 9 months after the introduction of POCTs in the region. Cases were women who had not been screened for syphilis during antenatal care and controls were women who had been screened. Patient-reported factors for being unscreened were examined using logistic regression to obtain odds ratios (ORs) and 95% confidence intervals (CIs).

**Results:**

160 consecutive unscreened and 327 screened women were recruited. Most women had good knowledge of syphilis (58.7% among unscreened women vs. 64.2% among screened; P = 0.24). Factors associated with failure to get screened were: attending antenatal care in a private health facility (adjusted OR, 11.09; 95% CI 5.48-22.48), previous adverse pregnancy outcome (adjusted OR, 1.98; 95% CI 1.22-3.23) and not being screened for HIV during the current pregnancy (adjusted OR, 2.78; 95% CI 1.50-5.13). The odds of being unscreened also increased with decreasing doses of intermittent preventive treatment for malaria in pregnancy received (P trend < 0.001) and decreasing education level (P trend = 0.02).

**Conclusion:**

Significant risk factors for not being screened, following the national rollout of syphilis POCTs, related to the type of health facility where antenatal care was received and some of the women’s personal characteristics. Targeting of private medical facilities to include syphilis POCTs and support other neglected public health interventions should be a priority.

## Background

Untreated maternal syphilis is associated with several adverse pregnancy outcomes, which are entirely preventable if the infection is detected and treated before the third trimester of pregnancy [1]. Although antenatal syphilis screening is a national policy in most sub-Saharan African countries, screening and treatment coverage remain typically low in many of these countries mainly due to technical and logistical challenges associated with reaginic testing [1,2]. Rapid point of care tests (POCTs) for syphilis which are of comparable performance to laboratory-based tests, can overcome most of these technical challenges [1,3] and have also been shown to be highly cost effective in sub-Saharan Africa [4]. In order to improve antenatal syphilis screening and treatment, these tests are recommended as screening tools in resource limited settings where most pregnant women do not have access to laboratories or syphilis testing [1,3]. In a recent multi-country study to assess the feasibility of introducing POCTs into antenatal clinics, POCTs were shown to increase antenatal syphilis screening and treatment coverage, resulting in the scale up of POCTs for antenatal syphilis screening in most of these countries [1].

Similar to other sub-Saharan African countries, antenatal syphilis screening had been poorly implemented in Ghana partly due to the above challenges [5]. However, following recent dramatic increases in reported treponemal infection prevalence among pregnant women, the Ghana Health Service in conjunction with its health development partners decided to scale up POCTs for antenatal syphilis screening in public health facilities throughout the country [6]. The current study is one of a number that were conducted to assess the rollout of syphilis POCTs in Ghana. Data pertaining to the factors associated with failure of pregnant women to screen for syphilis during antenatal care (ANC) following nationwide scale up of syphilis POCTs remains very sparse. We hypothesized that the major barriers to antenatal syphilis screening from the client’s perspective were likely to include: late booking, distance from the clinic, and lack of knowledge or offer of syphilis screening during antenatal care. The main aim of this study was to determine the factors associated with failure to be screened for syphilis during antenatal care in Ghana.

## Methods

We conducted an unmatched case–control study among women admitted for delivery in two public health district hospitals in the Ashanti Region of Ghana from August to October, 2010, the Manhyia District and St Patrick’s hospitals serving periurban and rural populations, respectively.

“Cases” were women who had not been screened for syphilis during ANC or did not have any syphilis results documented in the maternal (ANC) record booklet. “Controls” were women who had been screened for syphilis during ANC and had their results documented in the maternal record booklet. Two controls were selected consecutively on the same day for each case.

### Procedures

The research team visited the study sites early each morning before women were discharged from the hospital after delivery each day of the week except weekends and public holidays. Together with the midwives on duty, the research team reviewed the antenatal records of women who had delivered on the previous day(s) to identify eligible women for the study. For legal reasons, only women aged 18 and above were eligible, whilst women without antenatal records were excluded. Every eligible unscreened woman (“case”) and the next two eligible consecutive women who were screened for syphilis (“controls”) were selected. After giving informed consent, they were invited to participate in the study. All consenting women underwent a confidential interview in Twi or English using a pretested structured questionnaire and a review of their ANC record booklets.

Data on the women’s socio-demographic and reproductive health characteristics included: knowledge of exposure to recommended antenatal syphilis screening and treatment including partner notification and treatment, and treatment of babies of syphilis seropositive mothers at delivery; as well as other maternal and newborn interventions such as, prevention of mother to child transmission (PMTCT) of HIV, intermittent preventive treatment of malaria in pregnancy (IPT_p_) and tetanus toxoid administration were collected. Other risk factors for failure to screen for syphilis during antenatal care such as; gestational age at booking, cost and time of travel to the health facility, and type of health facility where ANC was received most were also explored.

Women’s knowledge of syphilis was assessed by evaluating their responses to nine questions on maternal syphilis including; mode of transmission, consequences, testing and prevention of mother-to-child transmission of syphilis. Each correct response attracted a score of “+1” while each “incorrect” or “undecided” (“don’t know”) response was assigned a score of “0”. The scores for each woman were summed and graded as follows; scores 0-3 = poor, 4-6 = average and 7-9 = good.

### Statistical power and analyses

Using Epi Info version 3.5.1 (Centers for Disease Control and Prevention, Atlanta, USA), we estimated that a sample size of 450 (150 unscreened women and 300 screened women) would have over 80% power to detect odds ratios (ORs) of 2.6, 3.7, 4.9 and 5.5 for late first ANC visit, history of previous STI, living far from antenatal syphilis screening service unit and lack of knowledge about the infection and antenatal syphilis screening, respectively, assuming that the proportion of screened women with these exposures were 10.9%, 10.1% , 23.2% and 38.9% respectively, as observed in a previous study from Mongolia [7]. This was the only available recent study which examined risk factors for failure to screen women for syphilis during ANC.

Categorical variables were compared according to syphilis screening status using the χ^2^, Fisher’s exact or Mann–Whitney rank sum tests as appropriate, while continuous variables were compared using the student t tests. Risk factors for being unscreened were reported using odds ratios (OR) and 95% confidence intervals (CIs) obtained by logistic regression. Univariable analysis was performed to examine the association of each explanatory variable with being unscreened and variables whose association reached statistical significance at P < 0.1 were included in a multivariable model. Significant explanatory variables including age as an a priori factor were added one at a time and those which remained independently associated with being unscreened at P < 0.1 were retained until all variables in the model were significant at P < 0.1. Excluded explanatory variables were retested in the final model one at a time to confirm lack of association. Tests for linear trend were performed for variables with more than two ordered categories. Likelihood ratio tests were used to assess the strength of association of each explanatory variable with the risk of being unscreened and linear trends.

The study was approved by the institutional review boards of the Ghana Health Service and Kwame Nkrumah University of Science and Technology, Ghana, and the London School of Hygiene and Tropical Medicine, United Kingdom.

## Results

### Study population

**Figure 1:**
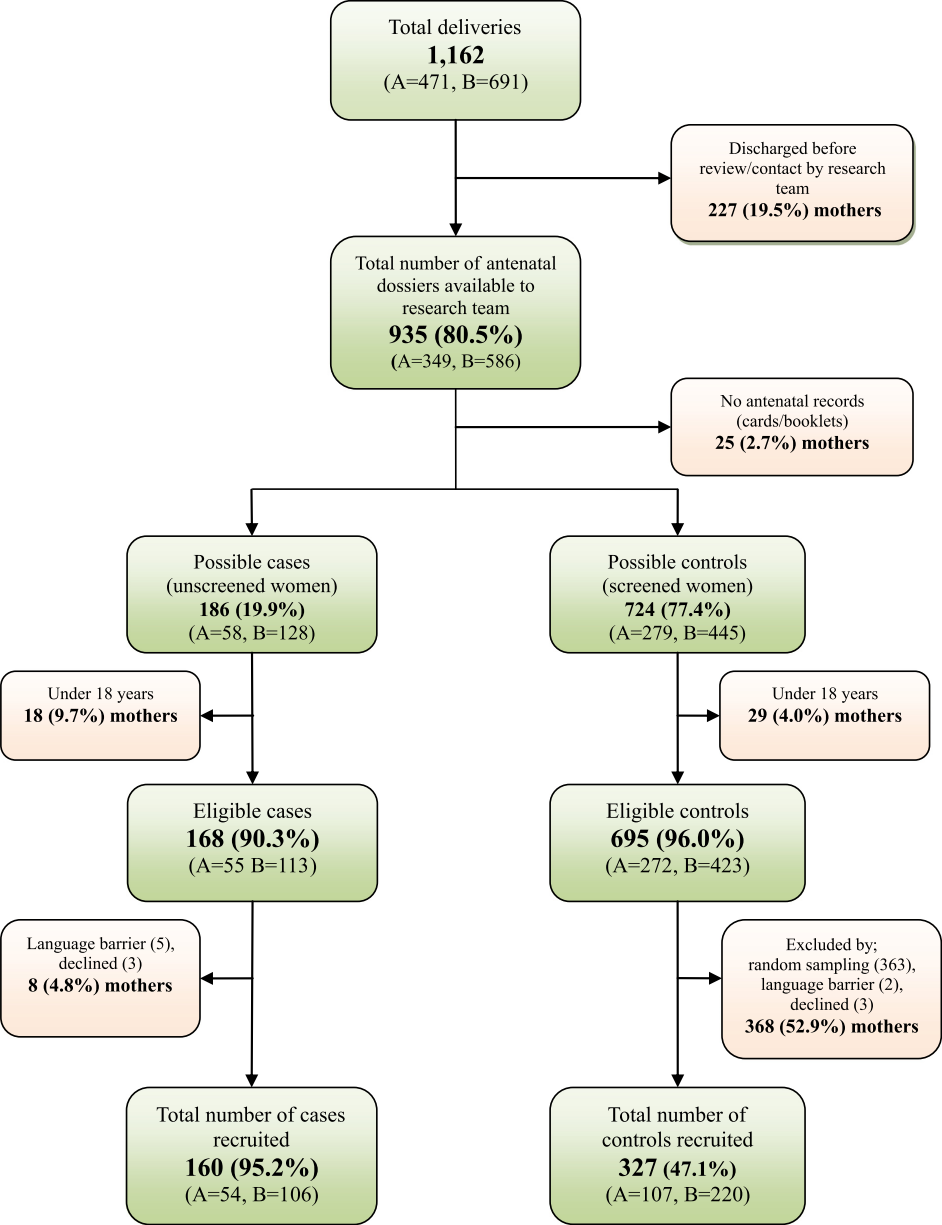
**Flow chart for recruitment of unscreened (cases) and screened (controls) women for antenatal syphilis.** (A = Manhyia District Hospital, B = St Patrick’s Catholic Hospital).

A total of 1,162 women delivered at the two hospitals within the study period, of whom 863 (74.3%) were eligible for inclusion into the study. Altogether, 160 (86.0%) of 186 unscreened women and 327 (45.2%) of 724 possible “controls” were recruited into the study (Figure 1). About two-thirds of the “cases” and “controls” were recruited from the larger St Patrick’s hospital.

### Background and reproductive characteristics of unscreened (“cases”) and screened (“controls”) women

The socio-demographic characteristics of “cases” (unscreened women) and “controls” (screened women) are shown in Table 1. Their age distributions were similar (mean ages 26.6 vs. 27.0 years-old). Women who had been screened for syphilis were more likely to have attended school (P = 0.04). Although the mode of travel and travel time to the health facility were not significantly different for cases and controls, unscreened women were more likely to have spent less than 0.50 Ghana cedis (~0.35 US$ in October 2010 [8]) as travel cost to the hospital compared to those who were screened for syphilis (P = 0.002).

**Table 1:** **Sociodemographic characteristics and antenatal syphilis screening status of women recruited at delivery**

**Characteristic**	Unscreened women N = 160 n (%)*	Screened women N = 327 n (%)*	**P-value**
**Age group, years**			0.83
18-19	22 (13.8)	33 (10.1)	
20-24	44 (27.5)	92 (28.1)	
25-29	45 (28.1)	99 (30.3)	
30-34	26 (16.3)	54 (16.5)	
35-46	23 (14.4)	49 (15.0)	
Mean (standard deviation[SD])	26.6 (6.2)	27 (6.2)	0.47
**Marital status**			0.15
Single	12 (7.5)	14 (4.3)	
Married/cohabiting	148 (92.5)	313 (95.7)	
**Religion**			0.14
Christian	112 (70.0)	249 (76.1)	
Muslim/other	48 (30.0)	78 (23.9)	
**Education**			0.04
No formal education	33 (20.6)	41 (12.5)	
Basic education	115 (71.9)	249 (76.2)	
At least secondary education	12 (7.5)	37 (11.3)	
**Occupation of woman**			0.45
Professional	3 (1.9)	13 (4.0)	
Vocational	43 (26.9)	75 (22.9)	
Trading/business	68 (42.5)	140 (42.8)	
Manual/farmer	13 (8.1)	39 (11.9)	
Unemployed	33 (20.6)	60 (18.4)	
**Occupation of spouse**			0.06
Professional	13 (8.1)	29 (8.9)	
Vocational	76 (47.5)	144 (44.0)	
Trading/business	31 (19.4)	95 (29.1)	
Manual/farmer	36 (22.5)	57 (17.4)	
Unemployed	4 (2.5)	2 (0.6)	
**Mode of travel to the clinic**			0.11
By foot	47 (29.4)	74 (22.6)	
Self/public transport	113 (70.6)	253 (77.4)	
**Average travel cost per visit to the clinic (Ghana cedis)**			0.002
<0.50	34 (30.4)	26 (14.3)	
0.5- < 1.00	55 (49.1)	146 (58.2)	
1.00+	23 (20.5)	69 (27.5)	
**Average travel time to the clinic**			0.32
<30 minutes	102 (63.8)	193 (59.0)	
30+ minutes	58 (36.2)	134 (41.0)	
**Registered with the National Health Insurance Scheme**			0.48
Yes	69 (43.1)	152 (46.5)	
No	91 (56.9)	175 (53.5)	
**Registered with the Free Maternity Care Scheme**			0.09
Yes	139 (86.9)	300 (91.7)	
No	21 (13.1)	27 (8.3)	

* Values are given as number (percentage) unless otherwise indicated.

The sexual and reproductive health characteristics of the women are shown in Table 2. Less than 5% of “cases” and “controls” reported having ever had a sexually transmitted infection (STI), genital ulcer disease or yaws (a non-venereal treponemal infection which cannot be distinguished from syphilis through blood tests). Compared to women who had been screened for syphilis during ANC, unscreened women were more likely to have attended private health facilities for ANC (P < 0.001), and less likely to have attended at least four ANC visits (P = 0.02), to have received HIV screening (P = 0.002) or three doses of IPT_p_ (P < 0.001). The association between syphilis screening status and any previous adverse pregnancy outcome (ie, spontaneous abortion, preterm delivery or stillbirth) was of borderline significance (29.4% vs. 21.7%; P = 0.06). Screened women had better knowledge of syphilis than unscreened women (64.2% vs. 58.7%), but the difference was not statistically significant. Late booking for first ANC visit was not significantly associated with being unscreened.

**Table 2:** **Reproductive and sexual health characteristics and antenatal syphilis screening status of women recruited at delivery**

**Characteristic**	**Unscreened women N = 160, n (%)**	**Screened women N = 327, n (%)**	**P-value**
**Number of pregnancies**			0.44
1-2	68 (42.5)	152 (46.5)	
3-4	49 (30.6)	104 (31.8)	
5-11	43 (26.9)	71 (21.7)	
Median (interquartile range)	3 (2–5)	3 (2–4)	0.54
**Ever had STI**			0.83
Yes	3 (1.9)	10 (3.1)	
No	154 (96.3)	310 (94.8)	
Does not know	3 (1.9)	7 (2.1)	
**Ever had genital ulcer disease**			0.82
Yes	5 (3.1)	9 (2.8)	
No	155 (96.9)	318 (97.2)	
**Ever had yaws**			0.59
Yes	3 (1.9)	5 (1.5)	
No	141 (88.1)	298 (91.1)	
Does not know	16 (10.0)	24 (7.3)	
**Condom use with regular partner**			0.86
Never	148 (92.5)	301 (92.1)	
Sometimes	12 (7.5)	26 (7.9)	
**Ever had spontaneous abortion**			0.10
Yes	30 (18.7)	43 (13.2)	
No	130 (81.3)	284 (86.8)	
**Ever had premature birth**			0.96
Yes	7 (4.4)	14 (4.3)	
No	153 (95.6)	313 (95.7)	
**Ever had stillbirth**			0.37
Yes	11 (6.9)	16 (4.9)	
No	149 (93.1)	311 (95.1)	
^**a**^ **Ever had any adverse pregnancy outcome**			0.06
Yes	47 (29.4)	71 (21.7)	
No	113 (70.6)	256 (78.3)	
**Facility where antenatal care was attended most**			<0.001
Public health facility	119 (74.4)	314 (96.0)	
Private health facility	41 (25.6)	13 (4.0)	
**Gestation at booking**			0.35
Early booking (up to 26 weeks)	150 (93.8)	313 (95.7)	
Late booking (after 26 weeks)	10 (6.2)	14 (4.3)	
**Number of antenatal care visits**			0.02
1-3	36 (22.5)	46 (14.1)	
4-16	124 (77.5)	281 (85.9)	
Median (interquartile range)	6 (4–8)	7 (5–9)	0.001
**HIV screening**			0.002
Yes	126 (78.8)	293 (89.6)	
No	34 (21.2)	34 (10.4)	
^**b**^ **Level of syphilis knowledge**			0.24
Poor (scores 1–3)	66 (41.3)	117 (35.8)	
Average-Good (scores 4–9)	94 (58.7)	210 (64.2)	
**Number of** ^**c**^ **IPT**_**p**_ **doses given**			<0.001
0	18 (11.2)	13 (4.0)	
1-2	75 (46.9)	118 (36.1)	
3	67 (41.9)	196 (59.9)	
**Number of tetanus toxoid doses given**			0.37
0	13 (8.1)	35 (10.7)	
1-2	147 (91.9)	292 (89.3)	

^a^Adverse pregnancy outcome: spontaneous abortion, preterm delivery or stillbirth.

^b^Total score for knowledge = 9: scores of 1-3 = poor; 4-6 = average; 7-9 = good.

^c^IPT_p_- Intermittent preventive treatment for malaria in pregnancy (using sulphadoxine-pyrimethamine).

Four women (1 of 126 unscreened and 3 of 293 screened women) with documented HIV results were HIV-1 seropositive; unscreened and screened women did not differ for their HIV sero-status (1% vs. 0.8%; P = 1.0).

### Factors associated with lack of syphilis screening during antenatal care

On univariable analysis (Table 3), unscreened women were more likely to have a lower education level, to have attended a private health facility for most of the current pregnancy, to have spent less than an average of 0.50 Ghana cedis as travel cost per visit to the health facility, to have had less than four ANC visits, to not have been screened for HIV and to have received less than three doses of IPT_p_ in the course of the current pregnancy. On multivariable analysis (Table 3), attending ANC in a private health facility hugely increased the odds of being unscreened at the time of delivery (adjusted OR, 11.09; 95% CI 5.48-22.48). Women who had experienced an adverse pregnancy outcome in a previous pregnancy were also more likely to be unscreened (adjusted OR, 1.98; 95% CI 1.22-3.23), as were those who had not been screened for HIV during the current pregnancy (adjusted OR, 2.78; 95% CI 1.50-5.13). The odds of being unscreened increased with decreasing number of doses of IPT_p_ received (P trend < 0.001) as well as decreasing level of education (P trend = 0.02).

**Table 3:** **Factors associated with women’s failure to be screened for syphilis during antenatal care in Ghana**

**Characteristic**	**Unscreened women N = 160, n (%)**	**Screened women N = 327, n (%)**	**Crude OR (95% CI)**	**Adjusted OR (95% CI)**
**Age group, years**			P = 0.83; P trend = 0.49	P = 0.94; P trend = 0.66
18-19	22 (13.8)	33 (10.1)	1.47 (0.77, 2.79)	1.13 (0.53, 2.38)
20-24	44 (27.5)	92 (28.1)	1.05 (0.64, 1.74)	0.86 (0.48, 1.53)
25-29	45 (28.1)	99 (30.3)	1	1
30-34	26 (16.3)	54 (16.5)	1.06 (0.59, 1.90)	0.86 (0.45, 1.65)
35-46	23 (14.4)	49 (15.0)	1.03 (0.56, 1.90)	0.86 (0.43, 1.71)
**Education**			P = 0.04; P trend = 0.01	P = 0.08**;** P trend = 0.02
No formal education	33 (20.6)	41 (12.5)	2.48 (1.12, 5.50)	2.84 (1.07, 7.53)
Basic education	115 (71.9)	249 (76.2)	1.42 (0.72, 2.83)	1.67 (0.72, 3.89)
At least secondary education	12 (7.5)	37 (11.3)	1	1
**Occupation of spouse**			P = 0.07	P = 0.10
Professional	13 (8.1)	29 (8.9)	0.85 (0.42, 1.73)	1.27 (0.56, 2.86)
Vocational	76 (47.5)	144 (44.0)	1	1
Trading/business	31 (19.4)	95 (29.1)	0.62 (0.38, 1.01)	0.63 (0.36, 1.10)
Manual/farmer	36 (22.5)	57 (17.4)	1.20 (0.72, 1.98)	1.23 (0.68, 2.25)
Unemployed	4 (2.5)	2 (0.6)	3.79 (0.68, 21.16)	5.11 (0.80, 32.47)
**Registered with the Free Maternity Care Scheme**			P = 0.09	
Yes	139 (86.9)	300 (91.7)	1	-
No	21 (13.1)	27 (8.3)	1.68 (0.92, 3.07)	-
**Ever had spontaneous abortion**			P = 0.10	
Yes	30 (18.7)	43 (13.2)	1.52 (0.91, 2.53)	-
No	130 (81.3)	284 (86.8)	1	-
**Ever had any adverse pregnancy Outcome**			P = 0.06	P = 0.006
Yes	47 (29.4)	71 (21.7)	1.50 (0.98, 2.31)	1.98 (1.22, 3.23)
No	113 (70.6)	256 (78.3)	1	1
**Facility where ANC was attended most**			P < 0.001	P < 0.001
Public health facility	119 (74.4)	314 (96.0)	1	1
Private health facility	41 (25.6)	13 (4.0)	8.32 (4.31, 16.08)	11.09 (5.48, 22.48)
**Average travel cost per visit to the clinic** (Ghana cedis)			P = 0.002; P trend = 0.002	
<0.50	34 (30.4)	26 (14.3)	2.83 (1.46, 5.51)	-
0.5- < 1.00	55 (49.1)	146 (58.2)	1.13 (0.64, 1.99)	-
1.00+	23 (20.5)	69 (27.5)	1	-
**Number of ANC visits**			P = 0.02	
1-3	36 (22.5)	46 (14.1)	1.77 (1.09, 2.88)	-
4-16	124 (77.5)	281 (85.9)	1	-
**HIV screening**			P = 0.002	P = 0.001
Yes	126 (78.8)	293 (89.6)	1	1
No	34 (21.2)	34 (10.4)	2.33 (1.38, 3.91)	2.78 (1.50, 5.13)
**Number of IPT**_**p**_ **doses given**			P < 0.001; P trend < 0.001	P = 0.002; P trend < 0.001
0	18 (11.2)	13 (4.0)	4.05 (1.88, 8.71)	3.72 (1.60, 8.68)
1-2	75 (46.9)	118 (36.1)	1.86 (1.25, 2.78)	1.78 (1.14, 2.79)
3	67 (41.9)	196 (59.9)	1	1

OR- Odds ratio; CI- confidence interval; ANC-Antenatal care; HIV-Human Immunodeficiency Virus; IPT_p_-intermittent preventive treatment of malaria in pregnancy.

### Characteristics and management of syphilis seropositive women

Nine women (2.8%) aged 20 to 31 years were seropositive for syphilis; seven (77.8%) of whom delivered at St Patrick’s Hospital and the remaining two at Manhyia District Hospital. All nine women had attended public health facilities for ANC and were HIV-negative. Six women had completed basic education, while the remaining three had had no formal education. Five (55.6%) women had average to good knowledge of syphilis and 4 (44.4%) were poorly knowledgeable about syphilis. Two women had had previous spontaneous abortions; no other previous adverse pregnancy outcome was reported. Five (55.6%) syphilis seropositive women had been treated for syphilis during ANC but only two babies were given prophylactic treatment for syphilis at birth, the remaining seven babies were offered treatment upon advice from the research team. No cases of congenital syphilis were diagnosed (by the attending staff) within the study period. Only two syphilis seropositive women were asked to invite their partners for counseling, testing and treatment, but the partners never honoured the invitations. We did not investigate whether these seropositive women had been screened for syphilis during a previous pregnancy.

## Discussion

This study identified maternal risk factors for the omission of syphilis screening during ANC in the context of a recent national rollout of treponemal POCTs in Ghana. The significant risk factors pertained to the health service which the women attended (private hospitals, and to some of the women’s personal characteristics (previous experience of adverse pregnancy outcome and their level of education).

Overall, most women had good knowledge of syphilis with no statistically significant differences between cases and controls. This contrasts with findings from a study in Mongolia where screened women had significantly higher knowledge scores for syphilis [7]. The findings in Ghana are not surprising since health education talks are given to all women attending ANC in both public and private clinics, and maternal syphilis is one of the key topics discussed, with strong encouragement for interactive discussions. In some facilities, especially where syphilis testing is performed in the laboratory, group counselling for syphilis testing is done at this stage, much similar to the “opt-out” approach of routine antenatal HIV screening [9]. These educational talks and counselling sessions are given in the local dialects, for example in Twi in the Ashanti region, to ensure that most pregnant women understand the issues being discussed. Poor knowledge of syphilis may be a result of not discussing syphilis during ANC as may have been the case in facilities not offering the tests, or when pregnant women reported late at their facility (thereby missing the health education component) or if the health talk had been poorly conducted. Notwithstanding the observed low syphilis seroprevalence among screened women, antenatal syphilis screening must be encouraged, as prenatal screening for syphilis has been found to be extremely cost effective, even at similar low prevalence [4,10].

The finding that pregnant women who attended private health facilities were at an increased risk of not being screened for syphilis is consistent with results from other studies in both developed and resource-constrained countries [7,11-14]. In resource-limited settings, most private facilities are usually smaller. Lack of test kits and limited capacities for performing syphilis tests tend to be major barriers to antenatal syphilis screening [7,11,12,14]. Most of the private health facilities attending to pregnant women in the study setting (peri-urban and rural areas of Ashanti region) were relatively small private midwife-managed maternity homes/clinics with limited infrastructure and staff. At the time of conducting this study, distribution of syphilis POCTs was limited to public health facilities and a few major private facilities. Pregnant women attending the smaller private facilities may not have been offered the test because the test kits were not available. The few women who got tested may have been referred by their providers to larger facilities for testing. While we did not investigate why pregnant women chose to pay more to access care in private health facilities and “receive less” (at least in terms of syphilis screening), other studies conducted in Tanzania and The Gambia suggest that seeking care in private facilities may be related to a better socio-economic status. Additionally, most pregnant women tend to be less satisfied with the physical environment, long waiting times, privacy during consultation, health care provider attitude and the provision of information or reassurance in most public facilities [15,16]. It is imperative that use of syphilis POCTs must be extended to these private health facilities if the goal of achieving universal access to antenatal syphilis screening and treatment is to be attained. While it may be easy to train private midwives to perform these tests, a key challenge however, may be monitoring that testing occurs and that the quality of testing provided by private midwives is maintained.

It is perhaps not surprising that women who were screened for HIV or who received IPT_p_ for malaria were more likely to be screened for syphilis, as all three interventions are usually administered in combination by midwives in most facilities, except in tertiary health facilities and few other facilities where doctors or medical assistants are required to order laboratory investigations or prescribe treatments. Our findings highlight the benefits and demonstrate the synergistic effects of providing an essential integrated package of services during ANC, as the uptake of one intervention will be influenced by the uptake of another antenatal intervention. Integrating maternal syphilis screening into ANC with established PMTCT programmes may not only be efficient and easier to implement [10,17,18], but also more cost-effective [19,20]. This can be facilitated by emerging technologies such as point-of-care dual or multiplex test kits combining tests for HIV, syphilis and other infections in a single cartridge [21]. The potential benefits of integration of ANC interventions are particularly obvious with the so-called “focused ANC” service provision in resource-limited settings, which can result in improvement in the quality of ANC, more efficient use of scarce resources by avoiding duplication of services, and making concurrent use of training, monitoring and supervision [18,19,22,23]. While taking advantage of integrated services, care must be taken not to overload limited staff or overburden already stretched health facilities beyond their capacities [11,23].

In agreement with previous studies [7,12,13], we observed an increasing risk of being unscreened with decreasing education levels. Unfortunately however, these women may also be at increased risk of syphilis infection since lower educational status appears to be an independent risk factor for congenital syphilis [24]. It is also possible that some of the women who experienced previous adverse pregnancy outcomes may have been seropositive for syphilis. These adverse pregnancy outcomes are known to be associated with maternal syphilis [25,26]. Moreover, routine antenatal syphilis screening was virtually non-existent prior to the rollout of syphilis POCTs.

Some women with positive syphilis serology and their babies were not treated, either because of ignorance or negligence on the part of the providers or lack of syphilis treatment (benzathine penicillin) in their facilities. It is unlikely that these women were screened for syphilis in their previous pregnancies. As observed in many prenatal services offering STI interventions in Africa [11,27-29], partner notification and treatment were poor in this study. It is unfortunate that most women were not asked to invite their partners, suggesting that post-test counselling was inadequate. Effective post-test counselling could have improved partner notification and treatment [27,28]. These seropostive women may also be at risk of re-infection since their partners could have been seropositive for syphilis. These findings indicate the need to ensure that there are clear and uniform guidelines for the screening and treatment of all pregnant women, and partners and babies of syphilis seropositive women in all facilities.

This study had a number of limitations. Our findings may be prone to selection bias since cases and controls were selected from healthcare facilities. Although ANC coverage was generally high (>90%), supervised delivery rate rates in the Ashanti region were much lower (42%) [30]. Women without antenatal records or access to ANC were excluded from the study: It is possible that risk factors for these women may be different from those who seek ANC or deliver at healthcare facilities. Women under 18 years were excluded due to legal reasons. However, younger pregnant women especially adolescents, may be less likely to use ANC services [31] and could be at increased risk of not being screened for syphilis during pregnancy. Women were considered to be unscreened if there was no documentation of a syphilis test result in their ANC record booklets. It is possible some women were screened but staff failed to record their test results, resulting in misclassification bias. This is however expected to be minimal as documentation in the ANC record booklet was generally good.

## Conclusion

Important risk factors for not being screened, following the national rollout of syphilis POCTs, related to the choice made by pregnant women of the health facilities where they seek antenatal care, which included places where even the basic ANC recommended package (HIV screening and IPT_p_ for malaria) was not implemented. Secondly, even where screening was carried out, important complementary interventions such as partner notification and prophylactic treatment of babies of syphilis seropositive women were not properly implemented either. Scaling up antenatal syphilis screening with syphilis POCTs to include private health facilities should be a priority, whilst this also presents with an opportunity to train their staff to deliver a comprehensive package of basic prenatal public health interventions. In the meantime, syphilis screening could be offered at delivery for women in public health facilities if their maternal records do not indicate that screening had been done, whilst management of mother and baby and improving partner notification should be strengthened.

## Abbreviations

POCTs: Point of care tests
OR: Odds ratio
CI: Confidence interval
ANC: Antenatal care
PMTCT: Prevention of mother to child transmission
HIV: Human Immunodeficiency Virus
IPT_p_: Intermittent preventive treatment of malaria in pregnancy
STI: Sexually transmitted infection
DFID: UK Department for International Development
